# Low levels of chicken body louse (*Menacanthus stramineus*) infestations affect chicken welfare in a cage-free housing system

**DOI:** 10.1186/s13071-024-06313-6

**Published:** 2024-05-14

**Authors:** Amy C. Murillo, Alireza Abdoli, Richard A. Blatchford, Eammon J. Keogh, Alec C. Gerry

**Affiliations:** 1 Department of Entomology, University of California, Riverside, CA USA; 2 Department of Computer Science and Engineering, University of California, Riverside, CA USA; 3 Department of Animal Science, Center for Animal Welfare, University of California, Davis, CA USA

**Keywords:** Ectoparasite, Poultry, Welfare, Sensors

## Abstract

**Background:**

The chicken body louse is an obligate ectoparasite of domestic chickens. Chicken body lice feed on feathers, and infestation with this louse is linked to decreases in egg production, hen weight, and feed conversion efficiency. However, it is unknown how chicken body lice impact egg-laying chickens in cage-free environments. Welfare and behavior metrics were collected from flocks of egg-laying chickens either infested with chicken body lice or left uninfested.

**Methods:**

In two trials, two flocks of cage-free commercial egg-laying chickens were infested with chicken body lice or maintained as uninfested controls. At three timepoints, behavior and welfare of all chickens was measured. On-animal sensors were used to quantify pecking, preening, and dustbathing behavior. Other animal-based welfare metrics included recording comb wounds and skin lesions.

**Results:**

Birds infested with chicken body lice exhibited significantly more preening behaviors than uninfested birds, even at low louse levels. Moderate or severe skin lesions were detected on birds that were moderately infested with chicken body lice while skin lesions were never detected on uninfested birds.

**Conclusions:**

The welfare of chickens was impacted by the chicken body louse, a chewing louse that primarily feather feeds. Evidence of skin lesions on infested birds suggests that lice may cause more damage to birds than previously thought, and further evaluation of louse economic damage is necessary.

**Graphical Abstract:**

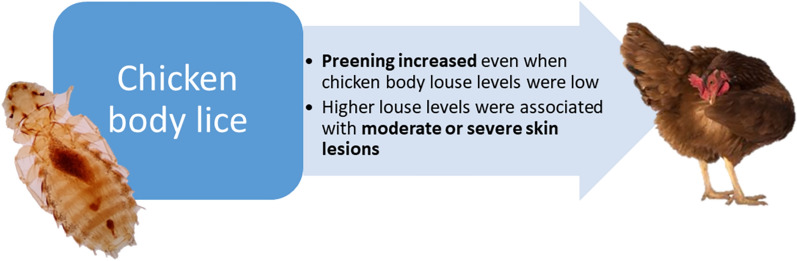

## Background

Commercial egg production in the USA is changing due to increasing animal welfare concerns. Just a decade ago, over 95% of egg-layers in the USA were housed in wire cages [1]. In response to legislation and animal welfare interests, there has been a substantial increase in the use of cage-free, free-range, or pasture-raised (“open-environment”) facilities for egg production [2–5]. Furthermore, noncommercial or “backyard” poultry production is increasing [6, 7], with these birds typically housed in cage-free facilities.

One concern with housing birds in cage-free environments is the increased potential for infestation by ectoparasites resulting from the greater exposure of birds to feces, contaminated soil, and wild birds or other animals compared with chickens housed in cages [1]. Therefore, housing changes will likely affect the diversity and prevalence of ectoparasites found in these flocks. Evidence of this change has been documented in non-commercial flocks in California, Idaho, Oregon, and Washington [8, 9], with greater ectoparasite diversity in backyard chicken flocks relative to commercial caged-chicken flocks [8]. The chicken body louse *Menacanthus stramineus* (Nitzsch) (Psocodea: Menoponidae) is the most common louse species on commercial and backyard poultry [8–10].

The chicken body louse is an obligate ectoparasite found on chickens and related poultry. These lice complete their entire 2–3-week life cycle on their chicken host [11], feeding on feathers and occasionally blood [12]. Chicken body louse infestations have been linked to decreased hen weight, egg production, and feed conversion [13]. However, few studies have investigated the effects lice have on chicken behavior or welfare in non-cage systems (e.g., [14, 15]). A limitation of these intensive behavior studies is the time investment required, as behavior studies often rely on the use of video recording and/or direct observation. This severely limits the number of animals that can be examined as well as the observation period for each animal, and it can be especially difficult to observe chicken behaviors associated with illness or disease, as sick birds are more prone to hiding these behaviors [16].

The use of technology, such as on-animal sensors, can increase the number and duration of behavioral observations without the confounding effects of human bias or interference (reviewed in Siegford et al. [17]). Use of on-animal sensors can also increase the number of individual animals tracked and the length of the tracking period, while also greatly increasing the sensitivity for detection of animal behaviors. Large amounts of data collection can provide more statistical power with less effort than studies reliant on direct observation or scoring video recordings. In the present study, we use on-animal sensors and visual assessment of chicken health to evaluate the behavior and welfare of chickens infested with chicken body lice compared with uninfested chickens.

## Methods

### Chickens

In each of the two trials, 48 beak-trimmed Hy-Line Brown laying hens were obtained from a local commercial poultry facility where they were beak-trimmed and vaccinated. Birds were housed at the Poultry Research Facility at the University of California Riverside (UCR) Agricultural Operations. The hens were 20 weeks old when enrolled in the study for both trial 1 and trial 2. Egg-lay began at approximately 19 weeks old, but production parameters were not recorded as part of the study. This study was approved by and conducted in accordance with the University of California Riverside Institutional Animal Care and Use Committee.

Two poultry houses (3.8 × 5.8 m) were divided in half to create four housing areas of equal size with each of the four housing areas containing a single flock of 12 birds. Each flock was provided with water dispensers, feed troughs, nest boxes, and had a bird density which met or exceeded US standards for cage-free production at the time of the study [18]. Straw bedding (5–10 cm in depth) was provided to birds throughout the study. Lights were kept on a 16:8 (L:D) h cycle. Each hen within a flock was uniquely marked with colored leg bands for individual bird identification.

### Lice

A colony of chicken body lice (CBL) is maintained on chickens held at UCR to serve as a source of these lice for research studies. Experimental birds were infested with CBL as described by Martin and Mullens [19]. Briefly, lice were gently brushed off CBL source birds into a white plastic dishpan where they could be readily seen. Then, study birds were infested with approximately 40 lice of mixed age and sex placed at the base of feathers in the vent region (underside) of each chicken to ensure a similar level of initial louse infestation on each bird. Birds were held for ~ 30 s to allow lice to settle on-host before returning the bird to the flock.

### Behavior

Three-axis accelerometers (“sensors”) (AX3, Axivity Ltd, UK) were used to record the direction and magnitude of acceleration as birds altered body position or moved within the poultry house. Sensors were placed in plastic “backpacks” (Hero 4 AHDBT-401 plastic case, Amazon.com, Seattle, WA, USA) affixed to the back of each bird using elastic bands stretched around the base of each wing [20]. Sensor data were collected at a rate of 100 Hz (approximately 100 readings/s).

A “behavior dictionary” was developed [20–22], which allowed for the classification of behaviors performed by birds from acquired sensor data. The sensor data that were used to build and test the behavior dictionary were collected from ten different birds (recorded for ≥ 4 h at a time) over the span of several months. Video recordings of test birds were synced with sensor output data and distinct behaviors were annotated by a single observer using ELAN open-access software (Max Planck Institute for Psycholinguistics, The Language Archive, Nijmegen, The Netherlands, v. 5.2, https://tla.mpi.nl/tools/tla-tools/elan/). Three chicken behaviors of interest were identified and are defined as (1) pecking: bringing the beak to the ground, striking at the ground; (2) preening: manipulating, rearranging, pulling, or smoothing body feathers by the beak; and (3) dustbathing: bird is in a sitting or lying position with feathers raised in a vertical wing-shake, including feather-ruffling and shaking [23]. Dustbathing and preening are both important for feather maintenance and thermoregulation [24]. Pecking was used as a proxy for foraging, a behavior associated with good welfare [25].

### Welfare

Welfare metrics adopted from the Welfare Quality Assessment® [26] were visually scored for each bird at three timepoints: week 1 (pre-infestation), weeks 7 or 8 (trial 1 or 2, respectively) and week 12. Each bird was visually examined from head to toe for abnormal condition of the eyes, beak, comb, keel, feet, toes, skin, and feathers. All metrics were scored as a 0, 1, or 2 (0, absent; 1, present and moderate; 2, present and severe).

### Experimental design

Trials were conducted in September–December 2018 (trial 1) and February–April 2020 (trial 2). Activities performed in each trial according to trial week are presented in Table 1. The four flocks were randomly assigned by house to a treatment (CBL-infested or control) with the two infested flocks receiving CBL at week 2 of each study trial. In trial 1, birds in the infested flocks also received CBL at week 6. Following the initial infestation at week 2, each bird was visually examined weekly for CBL by a single researcher (A.C.M.) for count consistency. CBL counts were performed by parting the feathers at the underwings, anterior keel, and vent region with all visible CBL life stages counted [27] to give a total CBL count per bird (CBL/bird). In both trials, behavior and welfare measures were recorded at week 1, before CBL were introduced to birds in the infested treatment flocks, at week 7 (trial 1) or week 8 (trial 2), and at week 12.

**Table 1:** Study schedule showing activity by week for each trial

Study week												
Study activity	1	2	3	4	5	6	7	8	9	10	11	12
Trial 1												
Welfare assessment	X						X					X
Sensor applied	X						X					X
CBL introduced		X				X						
CBL scored	X	X	X	X	X	X	X	X	X	X	X	X
Trial 2												
Welfare assessment	X							X				X
Sensor applied	X							X				X
CBL introduced		X										
CBL scored	X	X	X	X	X	X	X	X	X	X	X	X

Each trial used four flocks (*n* = 12 birds per flock). Chicken body lice (CBL) were added either to flocks 3 and 4 (trial 1) or flocks 1 and 2 (trial 2) at the weeks indicated, and the other two remained uninfested control flocks

### Statistical analyses

Statistical analyses were performed using SAS software (SAS Institute Inc., Cary, NC, 2012, v. 9.4), with PROC MEANS used to generate means and standard errors for CBL scores and behavior events.

A general linear model (PROC GLM) was used to determine whether bird behaviors differed across time for each flock or among flocks within a week, where the number of behaviors performed was the response variable and study week or flock was an independent variable. Means were separated by Tukey HSD for each behavior. Where behavior differences were found among flocks during the same week, PROC GLM was used to determine the effect of CBL count on bird behavior, where the number of behaviors was the response variable and the CBL count was an independent variable.

Nonparametric tests (PROC NPAR1WAY) were used to determine whether welfare metric scores varied by for each treatment group (infested or uninfested) from pre-infestation (week 1) to after CBL infestation (week 7/8 or week 12) with score 0 (absent) or 1–2 (present) as the response variable.

## Results

### Chicken body lice

**Fig. 1:**
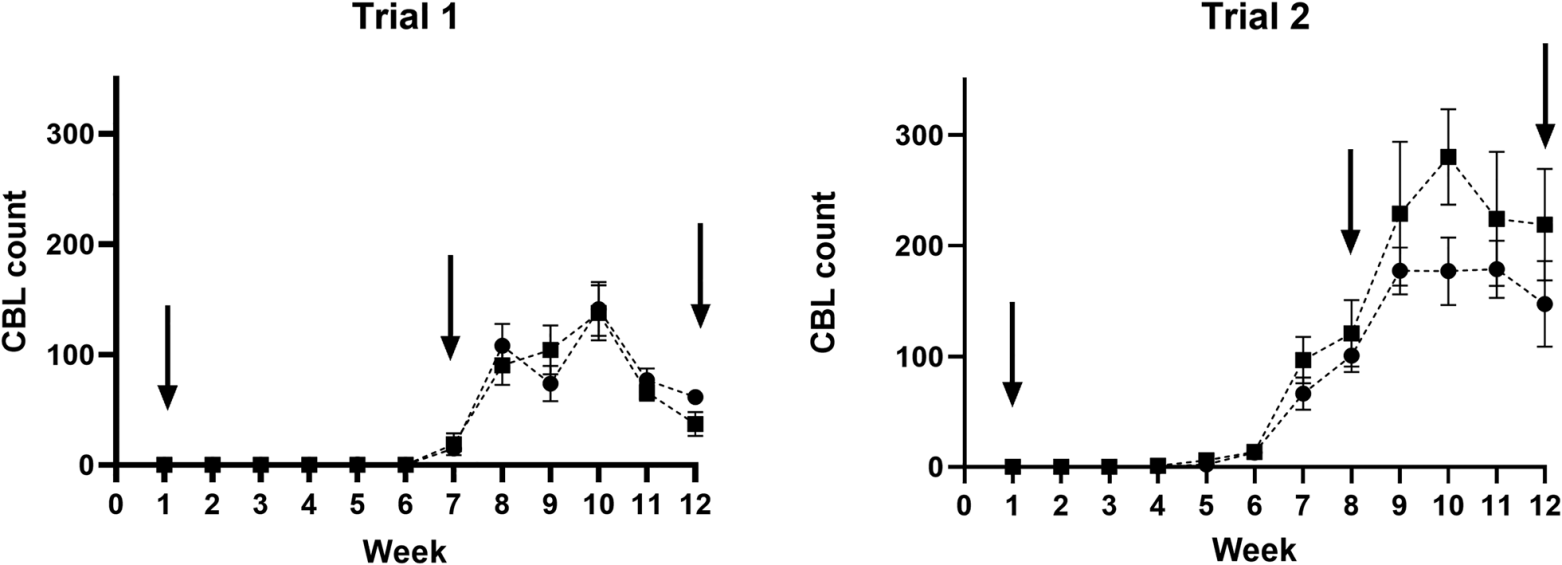
Chicken body louse/bird (means ± SE) in trial 1 and trial 2. In trial 1, flock 3 (circle) and flock 4 (square) were infested with chicken body lice during week 2 and again during week 6. In trial 2, flock 1 (circle) and flock 2 (square) were infested during week 2 only. The uninfested flocks in each trial (not shown) remained louse-free throughout both trials. Arrows indicate when behavior and welfare measures were recorded. *CBL* chicken body louse

During trial 1, the mean number of CBL (CBL count) on infested birds increased slowly, reaching a peak during week 10 (mean ± SE flock 3: 144 ± 24; flock 4: 138 ± 25) (Fig. 1). During weeks when behavior and welfare metrics were recorded, the CBL count for infested flocks was: week 1 (no CBL), week 7 (15 ± 3 and 19 ± 10), and week 12 (62 ± 6 and 37 ± 11) for flocks 3 and 4, respectively. During trial 2, CBL counts were overall much higher with peak CBL count for flock 2 occurring during week 10 (280 ± 43) and for flock 1 during week 11 (179 ± 26) (Fig. 1). During weeks when behavior and welfare metrics were recorded, the CBL count for infested flocks was: week 1 (no CBL), week 8 (101 ± 15 and 121 ± 30), and week 12 (147 ± 39 and 219 ± 51) for flocks 1 and 2, respectively. In both trials, CBL counts declined slightly after the peak through the end of the trial at week  12.

### Behavior

Pecking generally increased over time in all flocks in both trials, though a slight decrease in pecking was noted from week 7 to week 12 in trial 2. During trial 1, pecking was similar among all flocks during week 1 (*F* = 1.73; *df* = 3; *P* = 0.1746) and week 7 (*F* = 0.05; *df* = 3; *P* = 0.9852), but differed among flocks during week 12, with flock 1 (uninfested control) exhibiting more pecking than flock 4 (CBL-infested) (*F* = 5.85; *df* = 3; *P* = 0.0019). CBL count was not a significant factor affecting pecking (*F* = 1.87; *df* = 22; *P* = 0.0663). During trial 2 there were no significant differences observed among flocks during any week (week 1: *F* = 1.84; *df* = 3; *P* = 0.1539; week 8: *F* = 1.10; *df* = 3; *P* = 0.3607; week 12: *F* = 1.39; *df* = 3; *P* = 0.2582).

**Fig. 2:**
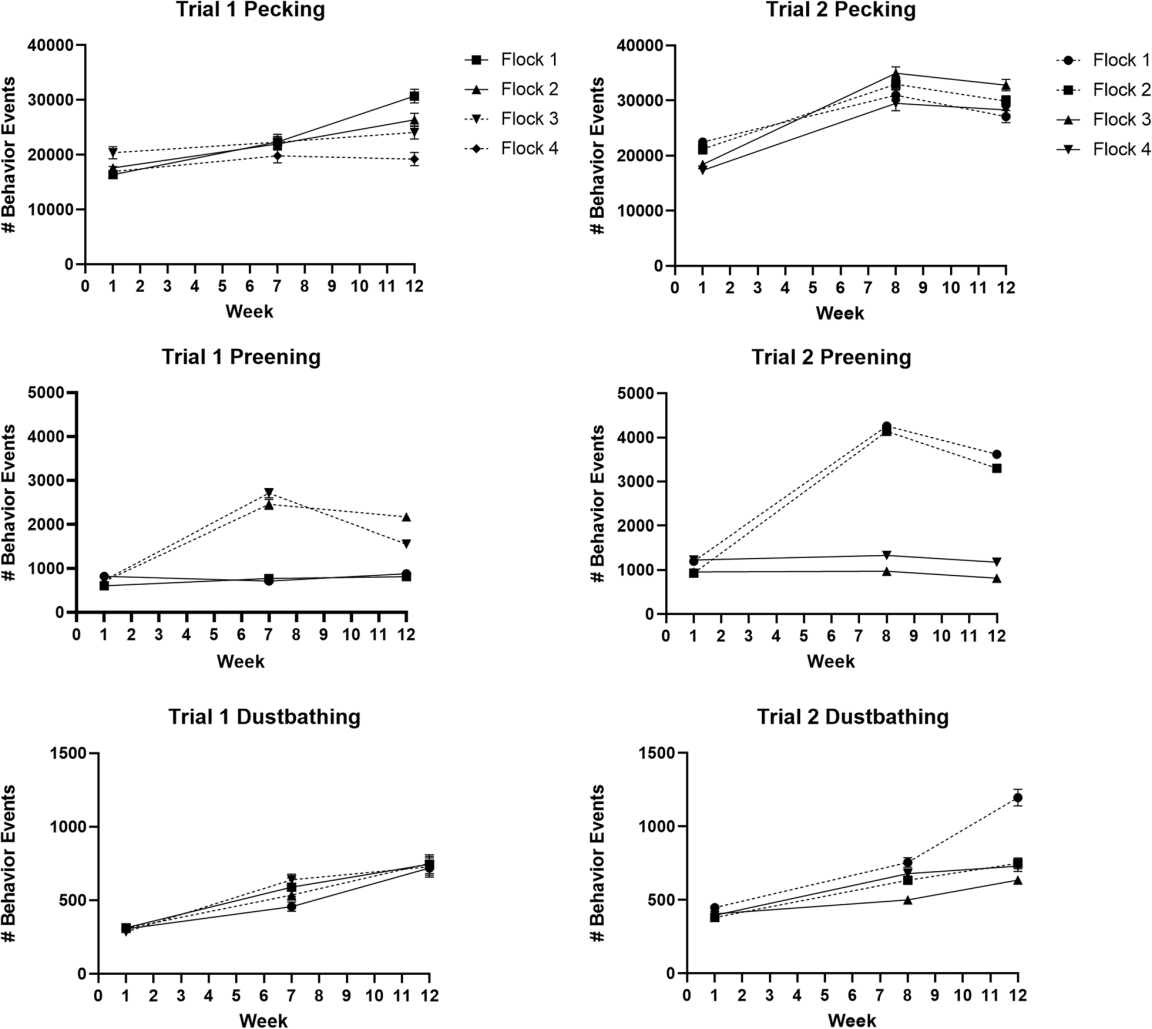
Behavior events (means ± SE) for all birds within each flock as recorded by on-animal sensors. Uninfested flocks are indicated by solid lines and louse-infested flocks are indicated by dashed lines. In both trial 1 and trial 2, CBL were present in infected flocks at week 7/8 and at week 12. *CBL* chicken body louse

Preening did not vary among flocks during week 1 (pre-infestation) for either trial (*F* = 1.16; *df* = 3; *P* = 0.3342 and *F* = 2.27; *df* = 3; *P* = 0.0936, for trials 1 and 2, respectively) (Fig. 2). However, preening was significantly greater in louse-infested flocks in trial 1 during week 7 (*F* = 79.05; *df* = 3; *P* < 0.0001) and week 12 (*F* = 27.49; *df* = 3; *P* < 0.0001) and in trial 2 during week 8 (*F* = 128.01; *df* = 3; *P* < 0.0001) and week 12 (*F* = 100.47; *df* = 3; *P* < 0.0001) with CBL count being a significant predictor of preening in both trial 1 (week 7: *F* = 28.58; *df* = 17; *P* < 0.0001; week 12: *F* = 9.12; *df* = 22; *P* < 0.0001) and trial 2 (week 8: *F* = 28.29; *df* = 22; *P* < 0.0001; week 12: *F* = 20.12; *df* = 24; *P* < 0.0001). In both trials preening was greater at the second observation time point (weeks 7 or 8) than at week 12 when CBL/bird had begun to decrease.

Dustbathing in both trial 1 and trial 2 gradually increased over time in all four flocks, with dustbathing recorded most often at week 12 (Fig. 2). In trial 1 there were no significant differences observed among flocks at any timepoint (week 1: *F* = 0.06; *df* = 3; *P* = 0.9789; week 7: *F* = 0.60; *df* = 3; *P* = 0.6174; week 12: *F* = 0.01; *df* = 3; *P* = 0.9978). In trial 2 there were no significant differences in dustbathing at week 1 or week 8 (week 1: *F* = 0.52; *df* = 3; *P* = 0.6730; week 8: *F* = 2.59; *df* = 3; *P* = 0.0648), but dustbathing by flock 1 occurred significantly more often at week 12 compared with the other flocks during this same week (*F* = 6.84; *df* = 3; *P* = 0.0007). The number of CBL was a significant factor affecting dustbathing (*F* = 3.93; *df* = 24; *P* = 0.0008).

### Welfare

Welfare metrics did not significantly differ between flocks of the same treatment in either trial and flocks were therefore pooled for analyses according to treatment (infested or uninfested). All comparisons were made to week 1 score, before lice were introduced to treatment groups. Few or no birds were found to have soiled feathers or eye, nose, feather, toe, or foot abnormalities, so these welfare metrics were not included in analyses.

**Fig. 3:**
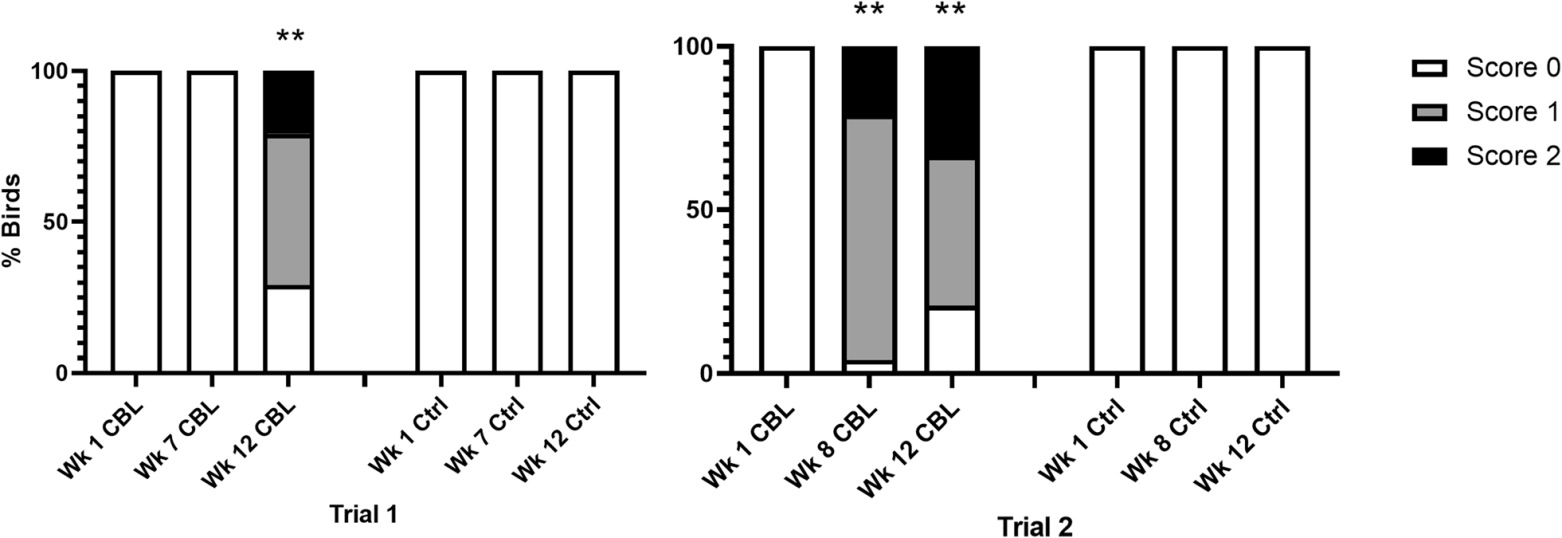
Skin lesion scores by week (wk) pooled by flock treatment (CBL, louse-infested flocks; Ctrl, uninfested control) in each trial. Bars indicate welfare scores for each metric as follows: white, 0 (normal); gray, 1 (moderate abnormality); black, 2 (severe abnormality). Skin lesions were not detected in uninfested flocks in either trial. Significant differences for presence/absence of skin lesions from week 1 (pre-infestation) to week 7/8 or week 12 are indicated by (**) *P* < 0.001. *CBL* chicken body louse

Skin lesions were never observed in uninfested control flocks or in treatment flocks during week 1, before birds were infested with CBL. Skin lesions were also absent from louse-infested flocks at week 7 in trial 1, but not at week 8 in trial 2. Skin lesions increased significantly from week 1 (pre-infestation) to week 12 in trial 1 and from week 1 to both week 8 and week 12 in trial 2 (T1 week 12: *χ*^2^ = 24.7752; *df* = 1; *P* < 0.001; T2 week 8: *χ*^2^ = 18.40; *df* = 1; *P* < 0.0001; T2 week 12: *χ*^2^ = 13.1429; *df* = 1; *P* = 0.0003) (Fig. 3).

**Fig. 4:**
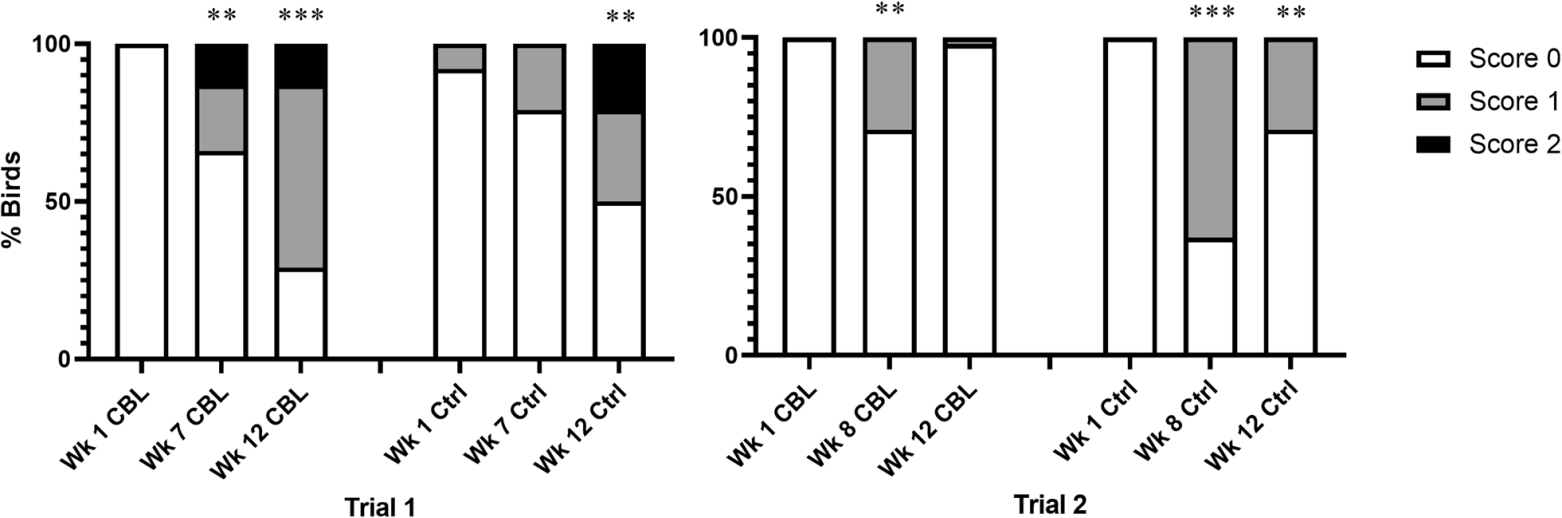
Comb wound scores by week (wk) pooled by flock treatment (CBL, louse-infested flocks; Ctrl, uninfested control) in each trial. Bars indicate welfare scores for each metric as follows: white, 0 (normal); gray, 1 (moderate abnormality); black, 2 (severe abnormality). Significant differences for presence/absence of skin lesions from week 1 (pre-infestation) to week 7/8 or week 12 are indicated by (**) *P* < 0.001; (***) *P* < 0.0001. *CBL* chicken body louse

Comb wounds were never observed in uninfested control flocks or in treatment flocks during week 1 (pre-infestation). In trial 1, louse-infested flocks exhibited significantly more comb wounds at week 7 and week 12 when compared with week 1 (week 7: *χ*^2^ = 9.3271; *df* = 1; *P* = 0.0023; week 12: *χ*^2^ = 25.0667; *df* = 1; *P* < 0.0001). In uninfested flocks, significantly more comb wounds were observed at week 12 compared with week 1 (*χ*^2^ = 10.4264; *df* = 1; *P* = 0.0012) but not at week 7 compared with week 1 (*χ*^2^ = 1.4739; *df* = 1; *P* = 0.2247). In trial 2, louse-infested flocks exhibited more comb wounds at week 8 compared with week 1 (*χ*^2^ = 8.0244; *df* = 1; *P* = 0.0046) but not at week 12 compared with week 1 (*χ*^2^ = 2.0435; *df* = 1; *P* = 0.1529). Uninfested flocks exhibited more comb wounds at week 8 and week 12 compared with week 1 (week 8: *χ*^2^ = 21.3636; *df* = 1; *P* < 0.0001; week 12: *χ*^2^ = 8.0244; *df* = 1; *P* = 0.0046) (Fig. 4).

## Discussion

In the present study, we observed peak CBL counts around 150 CBL/bird in trial 1 and 200–300 CBL/bird in trial 2. These CBL counts are comparable to recent experimental work [27], where small numbers of lice were introduced to beak-trimmed chickens. Previous studies have reported that chicken body lice infestations can reach up to 12,000 lice/bird on commercial chickens [28]. Characterizing chewing louse infestations is not straightforward, and factors such as bird size, breed, sex, or beak condition may impact numbers of lice per bird [27, 29]. Surveys of commercial flocks would provide insight into typical CBL infestation levels for commercial bird strains housed in production facilities.

Though lice were present at week 7 in trial 1, the counts were low (15–19 CBL/bird), and no skin lesions were detected suggesting louse numbers had not risen sufficiently to cause skin damage. Lice numbers increased substantially by week 8, but skin lesions were not assessed again until week 12. In trial 2, welfare metrics were performed a week later (at week 8) on the basis of results in trial 1, providing lice with an additional week to increase their numbers. At week 8 in trial 2, louse counts were relatively high (111 ± 16 CBL/bird), and skin lesions were present on chickens. This suggests that there is a CBL count threshold above which skin damage can be anticipated. Skin lesions were primarily observed to be in the underwing region of chickens where adult lice were observed to be chewing directly on the skin. This is an area where nymphal lice are also typically found [30] and is a location where lice may congregate to avoid host grooming [31, 32]. Throughout the study many adult and nymphal CBL observed had a visibly red abdomen providing evidence of blood feeding by these chewing lice. It is documented that Menoponidae lice in the clade Amblycera may occasionally feed on blood, perhaps due to chewing on developing pinfeathers which contain blood and sera (e.g., [12]). Crutchfield and Hixson [12] dissected CBL guts to examine their contents and found feather barbs and barbules and host nucleated red blood cells, however, they did not find evidence of host skin cells, suggesting that lice do not pierce the skin but instead get blood from young quill feathers. It is possible that host blood is a more important component of the CBL diet than previously known, and the chewing behavior observed by adults may provide blood for nymphal development, though this requires further study. Louse populations do decline naturally over time, possibly due to host-immune responses [27], which has also been observed to occur in response to chewing lice in other bird species [33]. Skin bruising in the vent region was occasionally observed in this study and has been observed on birds infested with chicken body lice (e.g., [32]), though this was not measured in the current study. Future work may try to deliberately measure the impact of skin bruising on bird health and welfare and relate that to louse infestations.

Comb wounds were observed in both louse-infested and uninfested flocks in each trial, with presence of wounds increasing after week 1. Since comb wounds were present on birds with CBL as well as on uninfested control birds, it appears that comb wounds are unrelated to the presence or activity of CBL. Other than aging of birds, no other obvious factors were noted among flocks over time to account for the increase in comb wounds from week 1 through week 12 of this study. A similar pattern was observed in a previous study with mite-infested birds [20], so the development of comb wounds over time may be typical in cage-free laying hen flocks.

While pecking and dustbathing behaviors increased over time in this study, these increases were not associated with CBL infestation as increases were noted in both CBL-infested and uninfested control flocks. This trend was similarly observed in chickens infested with a blood-feeding ectoparasite, the northern fowl mite (*Ornithonyssus sylviarum*) (Canestrini & Fanzango) (Acari: Macronysside) [20]. Additionally, the mean number of pecking events in the mite study was similar to the number of pecking events in the current study though the birds (same breed) in the mite study were slightly older. In the mite study, chicken behaviors were recorded using the same sensors and algorithms. The increase in pecking behavior over time may be an indicator of increased time spent feeding as birds grow.

Dustbathing generally increased over time in all flocks regardless of louse infestation, except in trial 2 when one CBL flock exhibited significant more dustbathing behavior. Dustbathing behavior is complicated and external factors can have a strong effect on expression of this behavior, changing its frequency and duration. Dustbathing may be socially facilitated [34] and variation among individuals may be influenced by social hierarchy [35]. Dustbathing is a circadian behavior that occurs infrequently, and data collection that is not restricted to a single time of day, such as in the present study, is best for sampling this type of behavior [36].

Preening behavior significantly increased when chickens were louse-infested, even when average louse levels were low during week 7 in trial 1. Significant differences were observed in the frequency of preening behavior between CBL-infested and uninfested flocks at all timepoints where lice were present. Murillo et al. [20] observed a similar increase in preening when chickens were infested with high numbers of northern fowl mite, though low mite numbers were not associated with increased preening. Vezzoli et al. [14] compared preening behavior of CBL-infested chickens that had trimmed or intact beaks, and beak-trimmed chickens spent significantly more time preening after infestation, with preening directed at the back, vent, and neck-chest area, all areas inhabited by CBL. In the current study, we could not detect where preening was performed, just the increase of the behavior over time. Interestingly, beak intact chickens showed no change in total time preening post-infestation [14]. Beak trimming can significantly impact a chicken’s ability to remove ectoparasites [8, 27], so it is possible that beak-intact birds were more efficient at removing irritating lice, thus keeping lice numbers low enough that increased preening time is not needed. In the USA, commercial chickens are still routinely beak-trimmed as young chicks to reduce feather pecking, cannibalism, and feed waste [37, 38].

Overall, in this study chickens exposed to chicken body lice exhibited similar behavior patterns to those reported for chickens exposed to the blood-feeding northern fowl mite [20]. One surprising difference is the relatively low levels of lice that resulted in behavior changes. In the current louse study, differences in preening were detected when lice were present compared with uninfested birds, even when louse counts were very low (such as trial 1). In the above mite study, differences in preening behavior were not observed until mite levels were numerically higher (> 500 mites/bird). This is surprising because northern fowl mites feed on host blood, often irritating the skin, while CBL typically feed on feathers. Perhaps the size of CBL (approximately five times larger than an adult northern fowl mite) increases the detection of lice by chickens.

Historically the chicken body louse has been the most common species of louse found in commercial poultry [10, 39, 40]. The reported economic effects of CBL have been conflicting, with some reports of no or insignificant impact on egg production (e.g. [28, 41]) and others reporting significant egg production losses (e.g., [13, 42]). Many factors may influence the effect of an ectoparasite on chicken production, including the age, sex, and breed of the host, previous parasite exposure, timing of infestation, and overall health of the animal. The increased frequency of preening and increased presence of skin lesions when birds were louse infested in the current study suggests that energy for growth or production may be diverted to removal of ectoparasites and perhaps also to increasing immune responses. While egg production and bird growth were not measured, future studies should track feed conversion efficiency and other economic indicators. It is possible that chewing louse damage to the host caused by skin chewing may be more prevalent and important than previously thought, and with more careful tracking economic thresholds may be developed for treatment decision making related to CBL infestations.

Currently the northern fowl mite is the most common and damaging ectoparasite of US poultry [40, 43, 44], though lice, including chicken body lice, may become more common in commercial poultry as cage-free, pasture raised, free-range, or slow-growing bird production increases due to increasing consumer-demand for organic eggs and meat. These types of production require birds to have access to the outdoors, and these open-environment farms provide more opportunities for birds to be infected with ectoparasites from wild birds or other animals, including several species of lice. Surveys of backyard and open-environment farms have shown high prevalence and diversity of lice is common on chickens, with co-infection (more than one species/bird) common [8, 9], Chambless et al.). It is unknown how chicken behavior, welfare, and production is influenced by co-infection with ectoparasites and/or endoparasites.

## Conclusions

Chicken body lice significantly increased chicken preening behavior and increased the frequency of skin lesions, even at low infestation levels. Future work is needed to better understand the impact of lice on chicken health and production factors to inform control efforts and producer decision making.

## Data Availability

The data supporting the findings of the study must be available within the article and/or its supplementary materials, or deposited in a publicly available database.

## Abbreviations

CBL: Chicken body louse
UCR: University of California Riverside

## References

[1] Lay DC, Fulton RM, Hester PY, Karcher DM, Kjaer JB, Mench JA (2011). Hen welfare in different housing systems. Poult Sci.

[2] Oberholtzer L, Greene C, Lopez E. Organic poultry and eggs capture high price premiums and growing share of specialty markets. US Department of Agriculture, Economic Research Service. 2016.

[3] Dawkins MS (2017). Animal welfare with and without consciousness. J Zool.

[4] Campbell DLM, Dyall TR, Downing JA, Cohen-Barnhouse AM, Lee C (2020). Rearing enrichments affected ranging behavior in free-range laying hens. Front Vet Sci.

[5] Cornell KA, Smith OM, Crespo R, Jones MS, Crossley MS, Snyder WE (2022). Prevalence patterns for enteric parasites of chickens managed in open environments of the western United States. Avian Dis.

[6] Pollock SL, Stephen C, Skuridina N, Kosatsky T (2012). Raising chickens in city backyards: the public health role. J Commun Health.

[7] National Animal Health Monitoring System (NAHMS) (2013). Urban chicken ownership in four U.S. cities.

[8] Murillo AC, Mullens BA (2016). Diversity and prevalence of ectoparasites on backyard chicken flocks in California. J Med Entomol.

[9] Chambless KN, Cornell KA, Crespo R, Snyder WE, Owen JP (2022). Diversity and prevalence of ectoparasites on poultry from open environment farms in the Western-United States of Washington, Idaho, Oregon, and California. J Med Entomol.

[10] Axtell RC, Arends JJ (1990). Ecology and management of arthropod pests of poultry. Annu Rev Entomol.

[11] Stockdale HJ, Raun ES (1965). Biology of the chicken body louse, *Menacanthus stramineus*. Ann Entomol.

[12] Crutchfield CM, Hixson H (1943). Food habits of several species of poultry lice with special reference to blood consumption. Fla Entomol.

[13] DeVaney JA (1976). Effects of the chicken body louse, *Menacanthus stramineus*, on caged layers. Poult Sci.

[14] Vezzoli G, Mullens BA, Mench JA (2015). Relationships between beak condition, preening behavior and ectoparasite infestation levels in laying hens. Poult Sci.

[15] Vezzoli G, Mullens BA, Mench JA (2015). Dustbathing behavior: do ectoparasites matter?. Appl Anim Behav Sci.

[16] Millman ST (2007). Sickness behaviour and its relevance to animal welfare assessment at the group level. Anim Welfare.

[17] Siegford JM, Berezowski J, Biswas SK, Daigle CL, Gebhardt-Henrich SG, Hernandez CE (2016). Assessing activity and location of individual laying hens in large groups using modern technology. Animals.

[18] United Egg Producers (UEP). Animal husbandry guidelines for U.S. egg laying flocks. 2017. https://uepcertified.com/wp-content/uploads/2021/08/CF-UEP-Guidelines_17-3.pdf. Accessed 9 May 2024.

[19] Martin CD, Mullens BA (2012). Housing and dustbathing effects on northern fowl mites (*Ornithonyssus sylviarum*) and chicken body lice (*Menacanthus stramineus*) on hens. Med Vet Entomol.

[20] Murillo AC, Abdoli A, Blatchford RA, Keogh EJ, Gerry AC (2020). Parasitic mites alter chicken behaviour and negatively impact animal welfare. Sci Rep.

[21] Abdoli A, Murillo AC, Yeh CCM, Gerry AC, Keogh EJ. Time series classification to improve poultry welfare. In: 2018 17th IEEE Int Conf Mach Learn Appl Icmla. 2018. p. 635–42.

[22] Abdoli A, Alaee S, Imani S, Murillo A, Gerry A, Hickle L, Keogh E. Fitbit for Chickens? In: Proc 26th ACM SIGKDD Int Conf Knowl Discov Data Min. 2020. p. 3328–36.

[23] Daigle CL, Banerjee D, Montgomery RA, Biswas S, Siegford JM (2014). Moving GIS research indoors: spatiotemporal analysis of agricultural animals. PLoS ONE.

[24] Olsson IAS, Keeling LJ (2005). Why in earth? Dustbathing behaviour in jungle and domestic fowl reviewed from a Tinbergian and animal welfare perspective. Appl Anim Behav Sci.

[25] Appleby MC, Smith SF, Hughes BO (1993). Nesting, dust bathing and perching by laying hens in cages: effects of design on behaviour and welfare. Brit Poultry Sci.

[26] Welfare Quality. Welfare Quality assessment protocol for poultry (broilers, laying hens). Welfare Quality Consortium, Lelystad, the Netherlands. 2009.

[27] Chen BL, Haith KL, Mullens BA (2011). Beak condition drives abundance and grooming-mediated competitive asymmetry in a poultry ectoparasite community. Parasitology.

[28] Stockdale HJ, Raun ES (1960). Economic importance of the chicken body louse. J Econ Entomol.

[29] Møller AP, Rózsa L (2005). Parasite biodiversity and host defenses: chewing lice and immune response of their avian hosts. Oecologia.

[30] Brown NS (1970). Distribution of *Menacanthus stramineus* in relation to chickens’ surface temperatures. J Parasitol.

[31] Brown NS (1972). The effect of host beak condition on the size of *Menacanthus stramineus* populations of domestic chickens. Poult Sci.

[32] Mullens BA, Chen BL, Owen JP (2010). Beak condition and cage density determine abundance and spatial distribution of northern fowl mites, *Ornithonyssus sylviarum*, and chicken body lice, *Menacanthus stramineus*, on caged laying hens. Poult Sci.

[33] Fairn ER, McLellan NR, Shutler D (2012). Are lice associated with ring-billed gull chick immune responses?. Waterbirds.

[34] Lundberg AS, Keeling LJ (2003). Social effects on dustbathing behaviour in laying hens: using video images to investigate effect of rank. Appl Anim Behav Sci.

[35] Shimmura T, Azuma T, Hirahara S, Eguchi Y, Uetake K, Tanaka T (2008). Relation between social order and use of resources in small and large furnished cages for laying hens. Brit Poult Sci.

[36] Daigle CL, Siegford JM (2014). When continuous observations just won’t do: developing accurate and efficient sampling strategies for the laying hen. Behav Process.

[37] Hester PY, Shea-Moore M (2003). Beak trimming egg-laying strains of chickens. J World’s Poult Sci.

[38] Mertens K, Löffel J, Baere KD, Zoons J, Baerdemaeker JD, Decuypere E (2009). Layers in aviary system: effects of beak trimming and alternative feed formulation on technical results and egg quality. J Appl Poult Res.

[39] DeVaney JA (1978). A survey of poultry ectoparasite problems and their research in the United States. Poult Sci.

[40] Hinkle NC, Hickle LA (1999). California caged layer pest management evaluation. J Appl Poult Res.

[41] Warren DC, Eaton R, Smith H (1948). Influence of infestations of body lice on egg production in the hen. Poult Sci.

[42] Gless EE, Raun ES (1959). Effects of chicken body louse infestation on egg production. J Econ Entomol.

[43] Mullens BA, Owen JP, Kuney DR, Szijj CE, Klingler KA (2009). Temporal changes in distribution, prevalence and intensity of northern fowl mite (*Ornithonyssus sylviarum*) parasitism in commercial caged laying hens, with a comprehensive economic analysis of parasite impact. Vet Parasitol.

[44] Murillo AC, Mullens BA (2017). A review of the biology, ecology, and control of the northern fowl mite, *Ornithonyssus sylviarum* (Acari: Macronyssidae). Vet Parasitol.

